# MEG measured delta waves increase in adolescents after concussion

**DOI:** 10.1002/brb3.2720

**Published:** 2022-09-02

**Authors:** Elizabeth M. Davenport, Jillian E. Urban, Christopher Vaughan, Jesse C. DeSimone, Ben Wagner, Mark A. Espeland, Alexander K. Powers, Christopher T. Whitlow, Joel D. Stitzel, Joseph A. Maldjian

**Affiliations:** ^1^ Advanced Neuroscience Imaging Research (ANSIR) Laboratory University of Texas Southwestern Medical Center Dallas Texas; ^2^ Department of Radiology University of Texas Southwestern Medical Center Dallas Texas; ^3^ Department of Biomedical Engineering Wake Forest School of Medicine Winston‐Salem North Carolina; ^4^ Virginia Tech—Wake Forest School of Biomedical Engineering Wake Forest School of Medicine Winston‐Salem North Carolina; ^5^ Department of Neurosurgery Wake Forest School of Medicine Winston‐Salem North Carolina; ^6^ Department of Radiology‐Neuroradiology Wake Forest School of Medicine Winston‐Salem North Carolina; ^7^ Clinical and Translational Science Institute Wake Forest School of Medicine Winston‐Salem North Carolina; ^8^ Childress Institute for Pediatric Trauma Wake Forest School of Medicine Winston‐Salem North Carolina; ^9^ Division of Pediatric Neuropsychology Children's National Health System Washington District of Columbia; ^10^ Division of Gerontology and Geriatric Medicine Wake Forest School of Medicine Winston‐Salem North Carolina

**Keywords:** concussion, delta waves, football, magnetoencephalography (MEG)

## Abstract

**Introduction:**

The purpose of this study is to determine if delta waves, measured by magnetoencephalography (MEG), increase in adolescents due to a sports concussion.

**Methods:**

Twenty‐four adolescents (age 14–17) completed pre‐ and postseason MRI and MEG scanning. MEG whole‐brain delta power was calculated for each subject and normalized by the subject's total power. In eight high school football players diagnosed with a concussion during the season (mean age = 15.8), preseason delta power was subtracted from their postseason scan. In eight high school football players without a concussion (mean age = 15.7), preseason delta power was subtracted from postseason delta power and in eight age‐matched noncontact controls (mean age = 15.9), baseline delta power was subtracted from a 4‐month follow‐up scan.

ANOVA was used to compare the mean differences between preseason and postseason scans for the three groups of players, with pairwise comparisons based on Student's *t*‐test method.

**Results:**

Players with concussions had significantly increased delta wave power at their postseason scans than nonconcussed players (*p* = .018) and controls (*p* = .027).

**Conclusion:**

We demonstrate that a single concussion during the season in adolescent subjects can increase MEG measured delta frequency power at their postseason scan. This adds to the growing body of literature indicating increased delta power following a concussion.

## Graphical Abstract

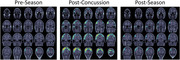

## 1 INTRODUCTION

American football is associated with the greatest number of mild traumatic brain injuries (mTBI), or concussions, out of all sports played in the United States (Daneshvar et al., 2011). Concussion can cause debilitating clinical symptoms and changes in neurocognitive measures (McCrory et al., 2012; McCrory et al., 2016). However, Clinical diagnosis of concussion can be challenging. Neuropsychological testing and symptom checklists, which are the primary method of diagnosis currently, can be subjective and nonspecific. Additionally, players may take steps to hide their symptoms, with estimates ranging from 33% to 78% of collegiate athletes reporting that they did not disclose their concussion (Delaney et al., 2015; Kerr et al., 2016). Returning to play with a concussion has been associated with long term detrimental and even fatal outcomes (d'Hemecourt, 2011), making an objective and quantitative method for clinical diagnosis and prognosis of concussion critically necessary. Conventional neuroimaging methods (i.e., CT scan) can provide nonbiased quantifiable results and may be used in the emergency department setting to rule out life‐threatening brain injury, however these gross‐level structural scans are usually unremarkable in cases of concussion. More advanced neuroimaging studies examining white matter integrity and neuronal electrical activity have demonstrated the possibility of potential microstructural and functional changes in the acute stages of mTBI (Alhourani et al., 2016; Churchill et al., 2017; Huang et al., 2012b; Lancaster et al., 2016; Mustafi et al., 2018). Most studies of structural changes, predominantly looking at white matter integrity, have shown compelling differences at the group level but have failed to translate these findings into clinical diagnostic metrics showing individual‐level differences.

Magnetoencephalography (MEG) has shown promise as a clinical diagnostic tool for concussion in individual patients. MEG is a noninvasive form of functional brain mapping that detects magnetic fields induced by neuronal electrical activity, with millisecond time scale resolution (Schwartz et al., 2010). Bundles of axons create intracellular currents from postsynaptic potentials, which have an associated magnetic field detectable by MEG (Baillet et al., 2001). Huang et al. (2012a) have previously measured functional changes using MEG in both civilian and military personnel with mTBI showing an increase in delta power after head injury both at the group level and for individual subjects. The delta spectrum consists of low‐frequency brain waves ranging from 1 to 4 Hz. These low‐frequency waves are considered pathologic in otherwise healthy, alert adults, and have been associated with brain lesions, Parkinson's disease, hypoxia, and states associated with abnormal or damaged brain tissue, in addition to mTBI (Knyazev, 2012). They have also been associated with schizophrenia, ADHD, and other psychiatric problems (Knyazev, 2012). These factors suggest MEG measured delta power is a compelling diagnostic and pronostic tool for concussion in adults. However, previous EEG and MEG studies have demonstrated that delta occurs normally in children and decreases into adulthood (Gasser et al., 1988; Gómez et al., 2013; John et al., 1980; Rodríguez‐Martínez et al., 2017). This decrease in delta power correlates with a reduction in gray matter volume with maturation. The reduction in gray matter is believed to be associated with a programmed reduction of synapses, or pruning (Huttenlocher & Dabholkar, 1997). Signals in the delta spectrum are thought to arise from synchronous activity between local cortical neurons. During pruning, a large number of these synapses are lost. This may be the cause of decreasing delta power into adulthood (Whitford et al., 2007). This dynamic change in delta power complicates the clinical diagnostic utility in pediatric patients. One study has evaluated MEG differences in pediatric patients with chronic concussion symptoms (Huang et al., 2020). However, no studies have yet to evaluate MEG in the acute setting in this population.

The purpose of this study is to determine if delta waves, measured by MEG, increase in adolescents during the acute phase of a sports concussion. We hypothesize that concussion in adolescents will result in an increase in delta waves in contradistinction to the natural tendency for delta waves to decrease at this age.

## 2 MATERIALS AND METHODS

All research procedures were approved by the institutional review board committee. Written informed parental consent and assent from participants were obtained. This study was Health Insurance Portability and Accountability Act‐compliant.

### 2.1 Protocol summary

The imaging Telemetry And Kinematic modeLing (iTAKL) study follows high school football players throughout a season of football in order to study the effect of subconcussive impacts on the brain. The football players receive preseason and postseason MRI and MEG imaging as well as cognitive testing. Preseason appointments were scheduled a few days to 1 month in advance of the first contact practice, and postseason scans were scheduled immediately after the season ended up to 1 month out. During the season, football players wore helmet‐embedded sensors known as the head impact telemetry system (HITS). The HITS data collection and MRI acquisition for this study are previously described in Davenport et al. (2016) as well as Urban et al. (2013) but are summarized here. As part of this larger study on subconcussive impacts, players who are clinically diagnosed with a concussion during the season also received postconcussion MRI and MEG imaging within 72 h of injury.

### 2.2 Subjects

Football players were recruited prospectively from local high school football junior varsity and varsity teams during the 2012–2019 football seasons. A total of 128 football players were recruited prospectively for the study. Subjects were excluded if they, or their parents, reported any of the following on the medical history questionnaire: (1) neurological disorders, (2) developmental disorders, (3) psychological disorders, (4) medications known to alter brain rhythms, (5) concussion within the last year, and (6) contraindication to MRI and/or MEG. An example of the online medical history questionnaire with a complete list of exclusion criteria is available in supplementary information. Board‐certified neuroradiologists reviewed all MRI scans as part of the study for clinically relevant abnormalities, and subjects with abnormalities were excluded from analysis. Ultimately, 50 players met inclusion criteria and completed the study with useable pre‐ and postseason imaging data sets. Eleven players with complete datasets who met inclusion criteria were diagnosed with a concussion during the season (mean age = 16.3). Three of those players did not return for postconcussion scanning within 72 h of injury and were excluded from the analysis. No players had loss of consciousness or other injuries at the time of concussion, nor were they hospitalized due to the concussion. All subjects followed return‐to‐play protocols and were scanned postseason. In addition, eight nonconcussed football players with no reported history of clinically diagnosed concussion were included in this analysis from the larger study. Subjects were identified that were age matched, ±1 month, and BMI matched, ±0.1, to the concussed players. If multiple matched subjects existed, the subject with the lowest subject identification number was chosen. To ensure this small group was representative of the average football player in the study, the head impact exposure metric for these players was compared to the average for all other nonconcussed athletes to ensure it was within one standard deviation of the mean (Urban et al., 2013). Player positions for both concussed and nonconcussed players are categorized into skill (quarterback, corner back, wide receiver, tight end, safety) or line positions.

Controls were recruited from local noncontact sports teams (swimming and tennis) and were excluded based on the criteria above as well as if they had any history of contact sports (i.e., football, lacrosse) or a history of concussion. Nine control subjects were recruited. Controls received baseline (preseason) and follow‐up (postseason) MRI and MEG imaging as well as cognitive testing at similar time points to football players. One control was excluded due to a clinically abnormal finding on MRI.

Ultimately, eight high school football players with a diagnosed concussion, eight high school football players without a diagnosed concussion, and eight age‐matched controls were included in this study. All subjects included in this study were male. MRI and MEG imaging were acquired for all concussed football players preseason, postconcussion, and postseason. The nonconcussed football players had pre‐ and postseason imaging. Age‐matched control subjects were scanned at two time points approximately 4 months apart.

### 2.3 Concussion diagnosis

Parents, players, and coaches were educated on the signs and symptoms of concussion prior to the season and were encouraged to report symptoms. An experienced certified athletic trainer evaluated all players preseason and was present at all practices and games to assess all athletes who presented with signs and symptoms of concussion. If a concussion was suspected, the subject was referred to our collaborating Concussion Clinic within 24–72 h of injury to confirm diagnosis by a Sports Medicine physician. All participants were still symptomatic at the time of their postconcussion scan.

Only the initial appointment at the Concussion Clinic, to confirm diagnosis of concussion for the study, was within the scope of the current study. After this initial visit, players and parents chose to continue care at the Concussion Clinic or follow‐up with their primary care physician. Therefore, data after the initial visit, specifically the return to play protocols they followed, are not available.

### 2.4 MEG acquisition and analysis

Continuous MEG signals sampled at a rate of 1200 Hz were recorded using a 273‐channel radial gradiometer whole‐head CTF Omega 2005 (VSM MedTech Ltd., Coquitlam, Canada) with an acquisition bandwidth of 0.25−150 Hz using all 273 cortical sensors and 29 reference sensors. Eight minutes of resting data were collected with eyes open.

Data sets for each subject were processed using Brainstorm (Tadel et al., 2011). MEG data were baseline corrected, band‐stop filtered (60 Hz), down‐sampled to 250 Hz, and band‐pass filtered to 1–100 Hz. Independent component analysis was performed and components representing eye blinks, cardiac artifact, and muscle artifacts were identified and removed using an in‐house machine learning algorithm and verified by an expert reviewer. Three minutes of clean, artifact free, data were selected by visual inspection for each scan for further processing. MEG data were coregistered with each subject's individual structural MRI scan, and data were source localized to a 5 mm isotropic grid using a minimum norm method. The delta frequency and total power, or energy/time, were computed for the 3 min of clean data for each voxel. The delta frequency power was normalized by the total power. The average whole‐brain power of the delta frequency and total power were computed for each scan. In the concussed football players, preseason delta power was subtracted from both concussion and postseason delta power. In nonconcussed football players, preseason delta power was subtracted from postseason delta power. For the control subjects, baseline delta power was subtracted from the 4‐month follow‐up scan. This process was repeated for the theta, alpha, beta, and gamma bands.

### 2.5 MRI acquisition and analysis

MRI data were acquired on a 3 Tesla Siemens Skyra MRI scanner using a high‐resolution 32 channel human head/neck coil (Siemens Medical, Malvern, PA). T1 anatomical images were obtained using a 3D volumetric MPRAGE sequence with isotropic resolution of 0.9 mm (TR = 1900; TE = 2.93; TI = 900; FA = 9; 176 slices). All subjects were fitted with fiducial markers placed on the nasion and bilateral preauricular regions to facilitate coregistration of MRI data with MEG. Structural T1 images were normalized to Montreal Neurologic Imaging (MNI) space using the Dartel high‐dimensional warping and the SPM8 (Ashburner & Friston, 2000) new segment procedure, as implemented in the VBM8 toolbox (http://dbm.neuro.uni‐jena.de/vbm.html). The T1 anatomic images were used for coregistration with the MEG data and normalization to standard Montreal Neurological Institute (MNI) space.

### 2.6 Statistics

The primary inference compared the change in delta power from preseason to postseason scans for the three groups of players using ANOVA, with pairwise comparisons between each group (3 tests total) based on Student's *t*‐test. This test was also repeated for baseline delta power, BMI, age, and time between pre‐ and postseason scans. Within the concussed group, we also compared the postconcussion and postseason delta power using a paired *t*‐test. This preseason to postseason comparison was repeated for all other spectral bands (theta, alpha, beta, and gamma).

A second ANOVA comparing concussion minus preseason delta power with postseason minus preseason delta power in controls and nonconcussed players was also conducted.

## 3 RESULTS

### 3.1 Primary results

Eight concussed football subjects, four skill and four line positions, were included. Demographics are outlined in Table 1 and detailed subject‐level information is provided in Tables S1.1–S1.3. They ranged in age from 14.6 to 16.9 years old, with a mean age of 15.8 (SD = 0.7) at preseason. The mean time between pre‐ and postseason scans was 130.1 days (SD = 24.8 days), and the mean time between pre‐ and postconcussion scans was 74.1 days (SD = 32.4). One of these players had a history of a previous clinically diagnosed concussion 4 years prior to the season. All players returned to play with clearance from a physician prior to the end of the season. Average postconcussion symptom inventory (PCSI) scores are provided in Table 2 and individual scores at each time point for the concussed players are included in Table S2. The eight nonconcussed football subjects, four skill and four line positions, ranged in age from 14.6 to 16.9 years old, with a mean age of 15.7 (SD = 0.7) at preseason, and mean time between pre‐ and postseason scans of 154.6 days (SD = 13.4 days). The eight control subjects ranged in age from 14.9 to 17.0 years old, with a mean age of 15.9 (SD = 0.9), and mean time between scans of 136.0 days (SD = 23.8 days). ANCOVA analysis showed no significant difference between groups for baseline delta power (*p* = .09, *F* = 2.7), BMI (*p* = .053, *F* = 3.4), age (*p* = .8, *F* = 0.23), or time between pre‐ and postseason scans (*p* = .09, *F* = 2.6). At baseline, the delta power between the controls and nonconcussed football players was significantly different (*p* = .024). The control group did have a significantly lower BMI than the concussed (*p* = .018) and nonconcussed (*p* = .035) football players. Time between scans was significantly different between the controls and nonconcussed football players (*p* = .37) as well as the concussed and nonconcussed football players (*p* = .016).

**TABLE 1:** Demographics

	Age (years)	BMI	Time between scans (days)
Concussed football players	15.8 ± 0.7	23.6 ± 2.7	Pre‐ to postconcussion: 74.1 ± 32.4** Pre‐ to postseason: 130.1 ± 24.8
Football players	15.7 ± 0.7	23.0 ± 2.6	154.6 ± 13.4
Controls	15.9 ± 0.9	20.1 ± 3.3	136.0 ± 23.8

** The time between pre and post‐concussion scans is significantly different than the time between scans for the football players and controls.

**TABLE 2:** Postconcussion symptom inventory (PCSI)

	PCSI preseason	PCSI postconcussion	PCSI postseason
Concussed football players	1.9 ± 3.8	26.0 ± 31.6	4.8 ± 5.6
Football players	2.5 ± 3.6	N/A	1.3 ± 2.4
Controls	1.3 ± 3.4	N/A	2.1 ± 3.4

**FIGURE 1:**
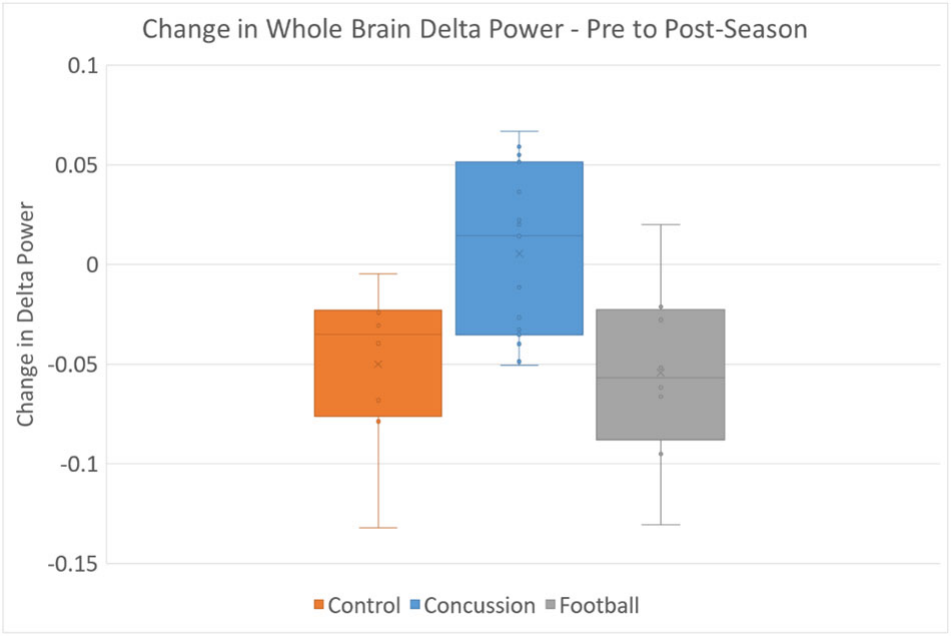
Change in delta power among groups. Based on pairwise comparisons, players with concussions had greater changes in delta wave power compared to nonconcussed players (*p* = .018) and controls (*p* = .027)

The mean pre‐ versus postseason changes in delta power varied among the three groups of athletes (overall *p* = .03, *F* = 4.1): concussed players had significant increases in their delta waves at postseason compared to the nonconcussed players (*p* = .018) and controls (*p* = .027). The change in delta power between pre‐ and postseason was not significantly different between the nonconcussed players and the controls (*p* = .85). These differences are shown in Figure 1 and summarized in Table 3. Additional subject‐level information is provided in Tables S3.1–3.3 and Figure S3. No significant differences between groups were found in other spectral bands.

**TABLE 3:** Comparison of change in delta values between groups at postseason time point

Comparison	*p* Value*
Control vs. concussed	.027
Control vs. typical football	.85
Concussed vs. typical football	.018

* Student's *t*‐test.

**FIGURE 2:**
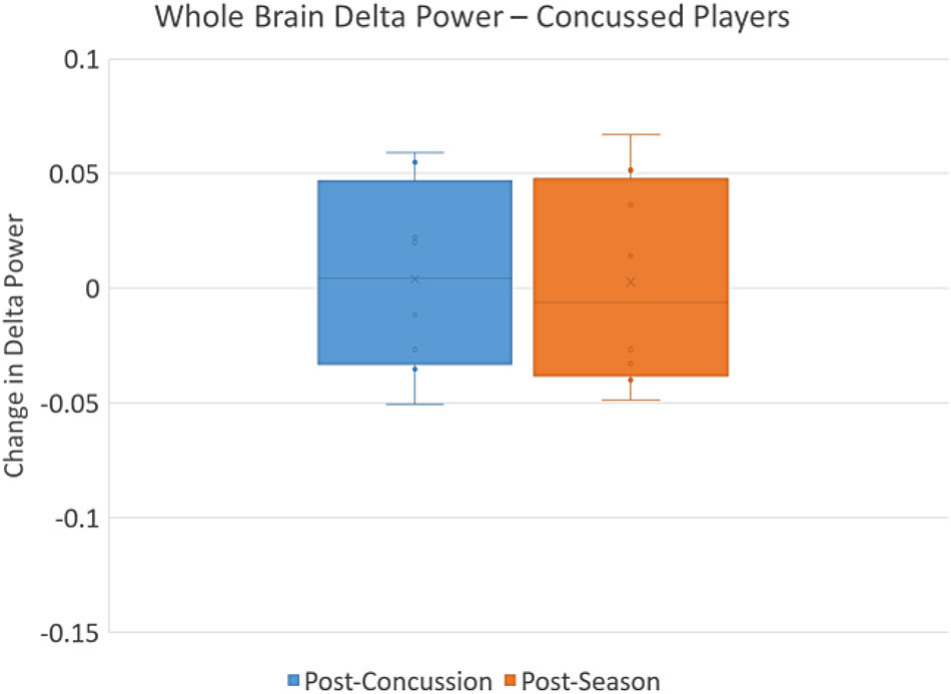
Change in delta power in concussed players. Based on paired *t*‐test the change in delta power postconcussion (compared to preseason) did not differ significantly from the change in delta power postseason in the concussed players

**FIGURE 3:**
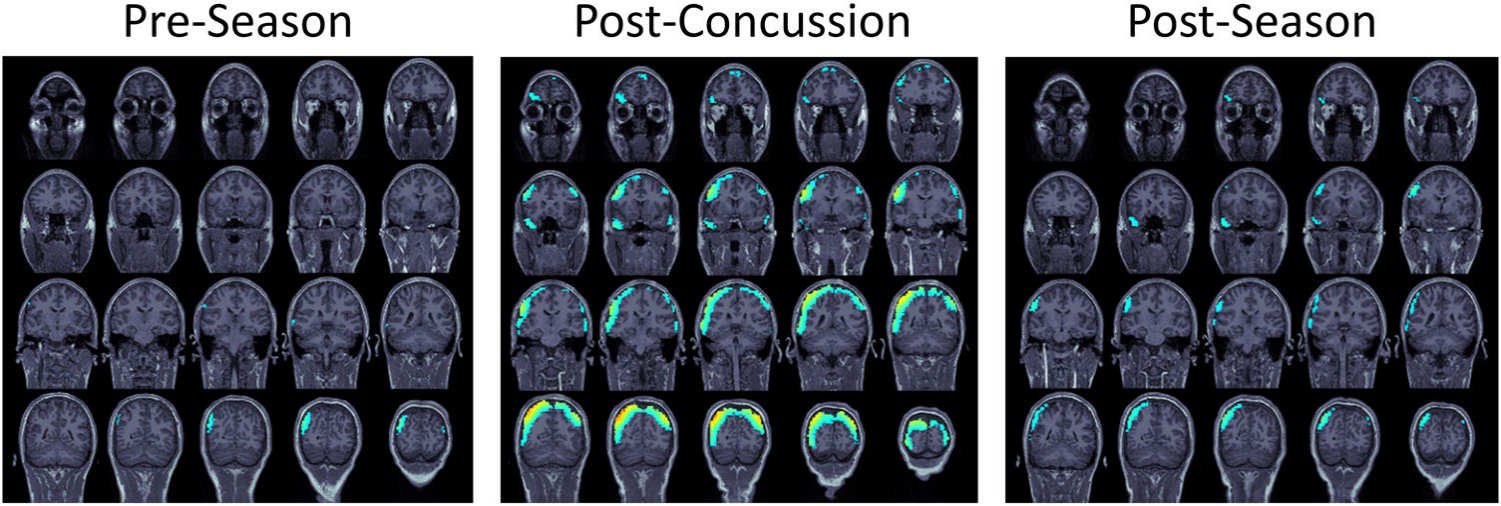
Delta waves of a concussed football player. Source localized delta power is shown in a single subject at the preseason, postconcussion, and postseason time points

Among concussed players, the difference in mean delta power from preseason was slightly greater postconcussion than at the season's end, but this difference did not reach statistical significance (*p* = .46) (Figure 2). A single concussed player's whole‐brain findings are visualized in Figure 3.

### 3.2 Secondary results

The secondary analysis comparing concussion minus preseason delta power in the concussed players with postseason minus preseason delta power in controls and nonconcussed players showed significant differences in delta power among the three groups of athletes (overall *p* = .022, *F* = 4.6): concussed players had significant increases in their delta waves at postconcussion compared to the nonconcussed players (*p* = .013) and the controls (*p* = .02). The nonconcussed players were not significantly different from the controls (*p* = .85). However, the time between scans for the preseason to postconcussion was significantly different from the time between scans for preseason to postseason in controls and nonconcussed players.

## 4 DISCUSSION

In this study, changes in MEG delta power in high school football players following clinically diagnosed concussion were compared to changes in delta power in an age and gender, matched control group over a similar timeframe. Whole‐brain delta power was significantly increased both immediately following a concussion and at postseason when a concussion was diagnosed during the season, compared to controls and nonconcussed players. Additionally, the increase in delta power at the time of concussion and did not vary significantly from the changes seen in the same group at postseason, suggesting that delta power remains elevated after athletes recover from clinical concussion symptoms.

Increased delta spectrum activity in EEG and MEG has long been associated with brain injury and disease (Conley et al., 2018; Lee & Huang, 2014; Modarres et al., 2017; Munia et al., 2017). However, the pathologic mechanism of increased delta activity in humans is still not well understood. Adult animal and adult human studies have shown that delta wave generation is related to white matter lesions, causing mechanical deafferentation (Ball et al., 1977; Gloor et al., 1977; Huang et al., 2009; Lukashevich et al., 1999). Diffuse axonal injury, resulting in cortical deafferentation, has been the suggested mechanism of delta waves in humans following concussion (Huang et al., 2014). Delta waves have also been induced in adult animal studies by injection of atropine (Castro‐Zaballa et al., 2019; Santucci et al., 1981; Schaul et al., 1978). Atropine causes deafferentation by blocking, or limiting, acetylcholine within the cholinergic pathway. Additionally, animal studies have demonstrated that traumatic brain injury causes dysregulation of the cholinergic system, and chronic reduction of acetylcholine (Dixon et al., 1996; Gorman et al., 1996). This would suggest that delta waves may not be the result of cell death or injury, but dysfunction at the synaptic level. Further studies are required to determine the mechanism of delta activity following TBI in humans, especially in pediatrics.

Regardless of the mechanism, delta spectrum activity is considered pathologic in alert adults (Steriade et al., 1990). However, children have naturally occurring delta spectrum activity when awake, which decreases with age. The age at which delta spectrum activity becomes pathologic is not precisely known, as it is based on individual maturation and development, but ranges from 13 to 22 years of age in EEG studies (Eeg‐Olofsson, 1980; Gasser et al., 1988; Matsuura et al., 1985; Whitford et al., 2007). The subjects in this study had an age range of 14.6 to 16.9 years. The control subjects had an age range from 14.9 to 17.0 years that was not significantly different from players. Given previous EEG literature, the expectation would be for the naturally occurring delta spectrum activity in this group to either remain constant or decrease. As seen in Figure 1, the control subjects did have decreased delta power over this time. However, there is limited literature on the MEG delta spectrum changes, or reliability, of normally developing children and adolescents especially over this short of a time frame. Further, data on the developmental age, hormone levels, and other factors associated with maturation, beyond age, were beyond the scope of this study. Therefore, raw MEG metrics were not compared between groups. Each subject was compared to their own baseline and the difference from baseline is reported. Overall, the finding of increased delta power, when compared to controls, is especially compelling given the tendency for delta power to decrease or remain constant in this age group.

### 4.1 Comparison with previous studies

Previous studies by Huang et al. and Lewine et al. have shown increased MEG measured delta power in military and civilian adults with chronic mTBI (Huang et al., 2016; Huang et al., 2014; Huang et al., 2012b; Huang et al., 2009; Lewine et al., 1999; Lewine et al., 2007). Huang et al. showed that, in adult subjects with chronic concussion, abnormal slow waves could be localized using MEG and the areas of the brain generating the slow‐waves correlated with postconcussive symptoms and DTI abnormalities (Huang et al., 2014; Huang et al., 2009). The studies by Huang et al. included a larger sample size of 84 concussed individuals, but subjects were adults and were only scanned once anywhere from 4 weeks to 5 years following concussion. The studies by Lewine et al. also studied adult subjects in the more chronic phases of concussion. Huang et al. (2020) recently studied 12 children with a history of mTBI using MEG and found differences in these children when compared to controls. These differences varied depending on the brain region and frequency band with the delta‐theta, alpha, beta, and gamma bands all showing both areas of increased power and decreased power compared to controls. Specifically, the delta‐theta band showed increases in the bilateral superior temporal lobe but decreases in the right superior frontal gyrus. It's important to note that these changes are documented 6 months after injury with few, if any, lingering symptoms. Additionally the children included in Huang et al.’s study were slightly younger (mean age of 12.58) than those included in this study. Additionally, these children all sought care at an Emergency Department (ED) after their initial injury and none of the subjects included in this study sought ED‐level care postinjury. In a more comparable population of adolescents (mean age 16 years) with sports related concussion less than 3 months prior to data collection, EEG showed significant global increases in the delta, theta, and alpha band power compared to controls as well as decreased beta power compared to controls (Munia et al., 2017).

**FIGURE 4:**
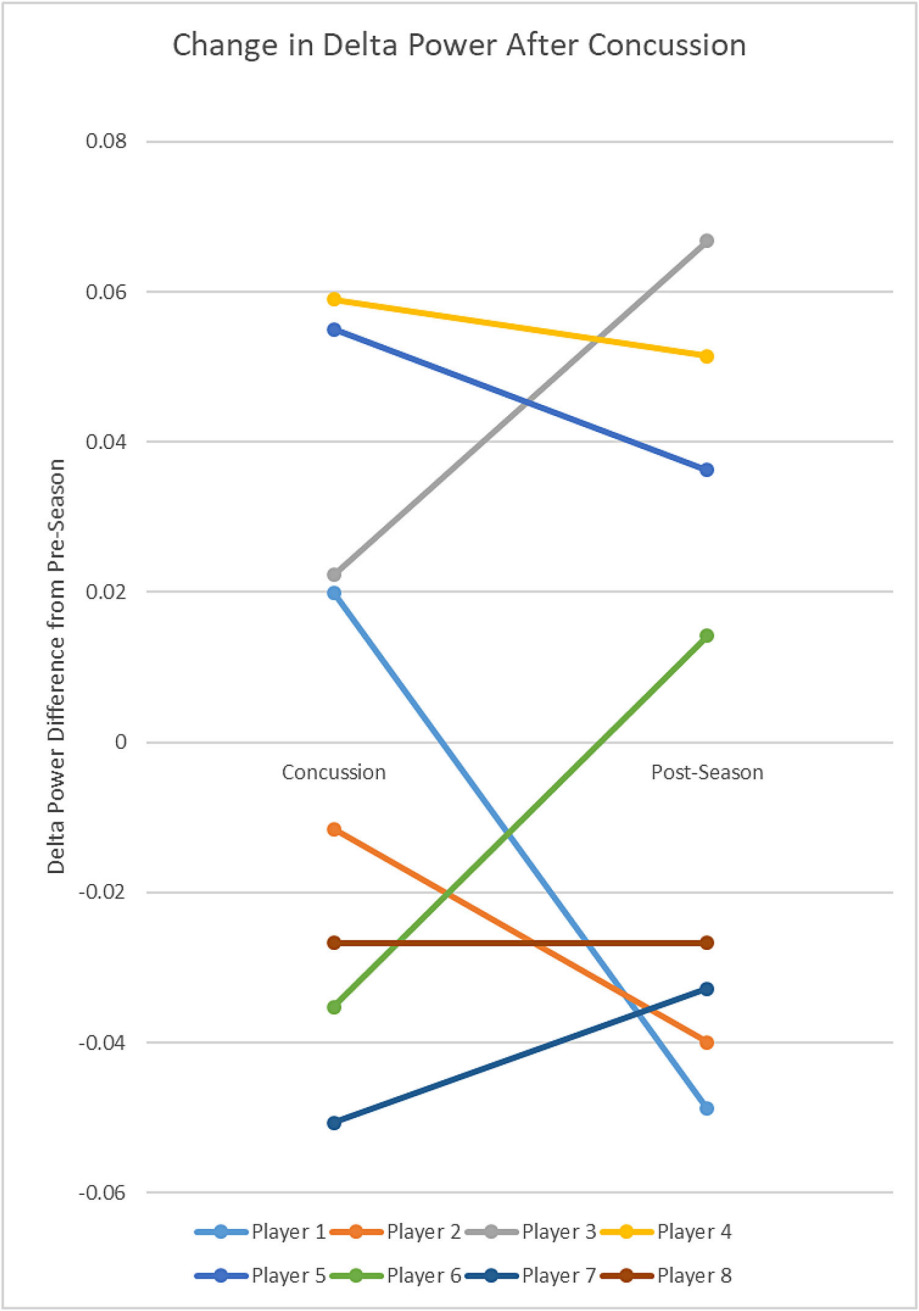
Change in delta power after concussion. The change in delta power from preseason is shown at postconcussion and at postseason for the 8 concussed football players. Each line represents a single subject. Three subjects had increased delta‐waves following a normal return‐to‐play protocol

The current study adds to this body of literature by studying an adolescent group in the acute phase of concussion as well as postrecovery. In particular, subjects were studied before concussion diagnosis, during the acute phase of concussion, and after symptoms had resolved. As shown in Figure 4, this allowed us to study the change in delta power after the subjects followed a return to play protocol, including final medical clearance from a physician. Most subjects had insignificant decreases in delta power after a return to play protocol was followed, compared to their immediate postconcussion time point. However, three subjects showed a slight increase in delta power when compared to their preseason time point. These three subjects did not have higher symptom scores compared to other concussed players at this time point, nor did they differ in terms of age, BMI, time between scans, or baseline delta power. The return to play protocol followed was beyond the scope of this study and should be investigated further in a larger longitudinal study as a potential factor. All control subjects, and 7/8 nonconcussed football players, showed decreases in delta power from pre‐ to postseason scans. Studies using EEG in adolescents and young adults have shown persistent EEG abnormalities following clinical recovery of sports‐related concussion (Barr et al., 2012; McCrea et al., 2010; Prichep et al., 2013). Longitudinal studies are required to determine the evolution of delta wave power following concussion and recovery.

### 4.2 Limitations

This study has several limitations. Our sample size and control group are relatively small and only includes males. However, it is one of the only studies to date of concussion in high school football that includes MEG and MRI. As discussed, concussion diagnosis is subjective and often relies on self‐report of symptoms. It is possible that concussion diagnosis in the typical football players were missed. Multiple factors were implemented to reduce this likelihood (see Section 2.3). Additionally, in the players with confirmed concussions, the return to play protocols and medical care after the initial diagnosis of concussion was beyond the scope of this study. The protocols followed could significantly affect the MEG data at the postseason scan and larger, longitudinal studies will be needed to further investigate recovery protocols. There are many factors that could potentially cause an increase or decrease in delta power. These factors include, but are not limited to maturation, developmental pruning stage, biomechanical effects of concurrent sports, and alertness. Studies of MEG measured delta power with normal development are limited in this age group. Given the direct correlation with clinical concussion and the neurologically normal sample used here, it is unlikely that the increase is due to other pathology. The relevance of these MEG changes after a concussion to long‐term outcomes is not known. Longitudinal studies are required to understand the long‐term influence of these changes on brain health and functioning.

## 5 CONCLUSION

We demonstrate that a single concussion in adolescent subjects can increase delta frequency power using MEG. The change in delta power may normalize after recovery from concussion, but longitudinal studies are required to determine the long‐term effects. This study adds to the growing body of literature indicating increased delta power following a concussion.

## CONFLICT OF INTEREST

The authors have no conflicts of interest related to this work.

### PEER REVIEW

The peer review history for this article is available https://publons.com/publon/10.1002/brb3.2720.

## Supporting information

Supplementary Information

## Data Availability

The data used in the submitted manuscript are available on the Federal Interagency Traumatic Brain Injury Research (FITBIR) database at https://fitbir.nih.gov/. The specific subjects used for this analysis are available on request from the corresponding author.
