# Supplementary material for: MEG measured delta waves increase in adolescents after concussion
Source: Brain Behav. 2022 Sep 2;12(9):e2720. doi: 10.1002/brb3.2720 (PMC9480906; doi:10.1002/brb3.2720)
Supplement: Supplementary file 1 — Supplementary Information [file BRB3-12-e2720-s001.docx]

Supplementary Information – Subject Level Values and Plots

S.1 - Baseline Values

Table S.1.1 – Baseline BMI

|  | Concussed | Control | Football |
| --- | --- | --- | --- |
| 1 | 25.1 | 21.9 | 26.4 |
| 2 | 26.4 | 26.5 | 21.3 |
| 3 | 21.4 | 20.0 | 20.8 |
| 4 | 24.0 | 21.0 | 22.7 |
| 5 | 22.1 | 18.4 | 24.9 |
| 6 | 19.9 | 16.0 | 22.8 |
| 7 | 27.6 | 16.8 | 26.1 |
| 8 | 22.0 | 20.0 | 19.0 |

Table S.1.2 – Baseline Age (years)

|  | Concussed | Control | Football |
| --- | --- | --- | --- |
| 1 | 15.7 | 15.0 | 17.0 |
| 2 | 16.1 | 17.0 | 15.6 |
| 3 | 16.5 | 16.2 | 15.3 |
| 4 | 14.6 | 17.1 | 15.5 |
| 5 | 15.6 | 15.1 | 15.8 |
| 6 | 15.3 | 15.0 | 15.5 |
| 7 | 15.7 | 16.7 | 14.7 |
| 8 | 17.0 | 15.7 | 16.4 |

Table S.1.3. - Time Between Pre- and Post-Season Scans (Days)

|  | Concussed | Control | Football |
| --- | --- | --- | --- |
| 1 | 158 | 131 | 156 |
| 2 | 117 | 135 | 171 |
| 3 | 157 | 131 | 161 |
| 4 | 147 | 110 | 156 |
| 5 | 137 | 117 | 136 |
| 6 | 115 | 120 | 133 |
| 7 | 113 | 176 | 165 |
| 8 | 139 | 168 | 159 |

S.2 – Concussed Individuals Information

Table S.2 – Concussed individuals Information

|  | Baseline PCSI | Concussion PCSI | Post-Season PCSI | Primary Compliant | Position | Concussion Hx |
| --- | --- | --- | --- | --- | --- | --- |
| Concussed 1 | 2 | 7 | 0 | Headache | Skill | None |
| Concussed 2 | 0 | 70 | 0 | Light/noise sensitivity | Line | 1 prior |
| Concussed 3 | 0 | 81 | 0 | Drowsiness | Skill | None |
| Concussed 4 | 1 | 6 | 0 | Emotional/Irritable | Line | None |
| Concussed 5 | 0 | 27 | 11 | Headache | Line | None |
| Concussed 6 | 0 | 5 | 0 | Concentration/Memory | Skill | None |
| Concussed 7 | 11* | 11 | 8 | Light/noise sensitivity | Line | None |
| Concussed 8 | 1 | 1 | 0 | Headache | Skill | None |

*Pre-season PCSI symptoms were sleeping more, being irritable, and difficulty remembering. None of these symptoms were endorsed post-concussion.

S.3 – Subject level Relative Delta Power

Table S.3.1 – Control Relative Delta Power

|  | Pre-Season | Post-Season |
| --- | --- | --- |
| Control 1 | 0.49281 | 0.4621 |
| Control 2 | 0.41185 | 0.40713 |
| Control 3 | 0.48312 | 0.4434 |
| Control 4 | 0.4701 | 0.40187 |
| Control 5 | 0.36872 | 0.34452 |
| Control 6 | 0.4553 | 0.37647 |
| Control 7 | 0.36982 | 0.34709 |
| Control 8 | 0.62109 | 0.48898 |

Table S.3.2 - Non-Concussed Players Relative Delta Power

|  | Pre-Season | Post-Season |
| --- | --- | --- |
| Player 1 | 0.37706 | 0.32523 |
| Player 2 | 0.40439 | 0.37648 |
| Player 3 | 0.41075 | 0.34455 |
| Player 4 | 0.4094 | 0.31422 |
| Player 5 | 0.3963 | 0.26564 |
| Player 6 | 0.42075 | 0.44076 |
| Player 7 | 0.33048 | 0.30925 |
| Player 8 | 0.39692 | 0.33521 |

Table S.3.3 - Concussed Players Relative Delta Power

|  | Pre-Season | Concussion | Post-season |
| --- | --- | --- | --- |
| Concussed 1 | 0.38643 | 0.4064 | 0.33763 |
| Concussed 2 | 0.44548 | 0.43393 | 0.40551 |
| Concussed 3 | 0.40575 | 0.42803 | 0.47253 |
| Concussed 4 | 0.29697 | 0.35595 | 0.3484 |
| Concussed 5 | 0.51192 | 0.5669 | 0.54821 |
| Concussed 6 | 0.40109 | 0.36581 | 0.41525 |
| Concussed 7 | 0.40758 | 0.35691 | 0.3748 |
| Concussed 8 | 0.37234 | 0.34563 | 0.34563 |


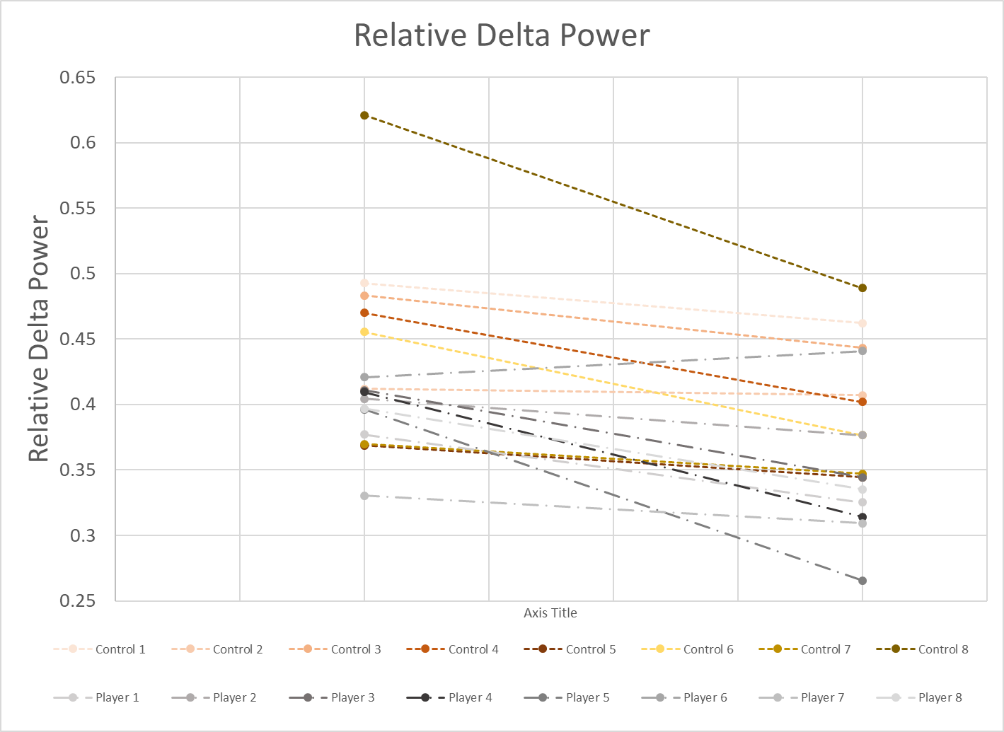


Figure S.3 Pre- to Post-Season Delta Power for individual controls and non-concussed football players. One football player showed increased delta power from pre-season to post-season. This player did not have a significantly different head impact exposure from the group.
